# Enhanced Expression of N-Cadherin, but Not of E-Cadherin, in Cutaneous Squamous Cell Carcinoma in Comparison to Basal Cell Carcinoma

**DOI:** 10.3390/cancers16244247

**Published:** 2024-12-20

**Authors:** Joanna Pogorzelska-Dyrbuś, Danuta Nowicka-Suszko, Aleksandra Piotrowska, Zdzisław Woźniak, Piotr Dzięgiel, Jacek C. Szepietowski

**Affiliations:** 1 “Estevita” Specialist Medical Practice, 43-100 Tychy, Poland; jpogorzelskadyrbus@gmail.com; 2 University Centre of General Dermatology and Oncodermatology, Wroclaw Medical University, 50-367 Wroclaw, Poland; danuta.nowicka-suszko@umw.edu.pl; 3 Department of Human Morphology and Embryology, Division of Histology and Embryology, Wroclaw Medical University, 50-367 Wroclaw, Poland; aleksandra.piotrowska@umw.edu.pl (A.P.); piotr.dziegiel@umw.edu.pl (P.D.); 4 Department of General and Experimental Pathology, Wroclaw Medical University, 50-367 Wroclaw, Poland; zdzislaw.wozniak@umw.edu.pl; 5 Department of Dermato-Venereology, 4th Military Hospital, 53-114 Wroclaw, Poland; 6 Faculty of Medicine, Wroclaw University of Science and Technology, 50-370 Wroclaw, Poland

**Keywords:** E-cadherin, N-cadherin, Ki67, BCC, SCC

## Abstract

Background: Adhesion molecules including E-cadherin and N-cadherin have been proven to contribute to the carcinogenesis process. It has been demonstrated that an increased expression or appearance of N-cadherin, as well as a reduction in the expression of E-cadherin, are documented in many cancers, often leading to the loss of intercellular adhesion and acquisition of a more invasive or even metastatic mesenchymal phenotype. The aim of this study was to assess the expression of E-cadherin and N-cadherin, as well as markers of proliferation Ki67 in basal cell carcinoma (BCC) and squamous cell carcinoma (SCC). Methods: A total of 123 tumor paraffin specimens, including 73 BCC and 50 SCC cases, were obtained from multiple anatomical locations. The expression of E-Cadherin and N-Cadherin, including the percentage of stained cells, was assessed using a four-grade scale, with Ki-67 assessed on the five-grade scale. Results: A significantly higher expression of N-cadherin was observed in SCC compared to BCC, with 14% of SCC cases having a more than 50% expression of N-cadherin, and 10% with 26–50% expression, in comparison with 2.7% and 8.2% in BCC, respectively (*p* < 0.001). No significant differences were observed with regard to E-cadherin expression between SCC and BCC. Conclusions: Our results suggest that N-cadherin expression might contribute to the acquisition of the mesenchymal phenotype, SCC, when compared with BCC, with a high expression of E-cadherin in both tumors explaining their overall low rate of metastases; however, further research on the role of adhesion molecules in these tumors is needed.

## Simple Summary

In this manuscript, the expression of adhesion molecules, E-cadherin and N-cadherin, in two common skin cancers: basal cell carcinoma and squamous cell carcinoma, were analyzed and compared. The aim of the manuscript was to evaluate the differences in protein expression between these two cancer types and how that might relate to their likelihood of aggressiveness. In the manuscript, a significantly higher expression of N-cadherin, but not of E-cadherin, was observed in SCC compared to BCC. Thus, this might suggest that N-cadherin expression contributes to the acquisition of mesenchymal phenotype in SCC when compared with BCC, with a high expression of E-cadherin in both tumors explaining their overall low metastatic potential.

## 1. Introduction

Non-melanoma skin cancers (NMSC) are the most frequently diagnosed malignancies and their prevalence has increased dramatically over the last 30 years, generating significant medical and financial problems worldwide [1]. Basal cell carcinoma (BCC) and squamous cell carcinoma (SCC) represent 99% of all NMSC with rare occurrence of metastases varying from 0.0028% to 0.55% and 0.1–9.9% for BCC and SCC, respectively [2,3]. The explanation for the low, although different, rates of metastases in both cancers is still under investigation, and the mechanisms of metastasis formation are still insufficiently known [4,5]. In general, the process of cancer metastasis consists of the detachment of tumor cells from the primary site, their intravasation into the blood, extravasation into distant tissues and the formation of secondary lesions. The first important stage in the process of metastasis for the detachment of cells from the solid tumor site appears to rely on the loss of their epithelial phenotypes, weakening of the cell–cell adhesion and the acquisition of mesenchymal phenotypes [6,7]. Normal epithelial integrity results from the interaction between keratinocytes, and this is mediated by adhesion molecules; of these, the most important are Ca^2+^-dependent classical cadherins. [8]. Among them, E-cadherin or epithelial cadherin also known as CDH1 is the best described. It consists of a large extracellular domain that is responsible for the homotypic cadherin–cadherin interaction, the transmembrane domain and an intracellular domain that is closely associated with the cytoskeleton [9]. E-cadherin, by its cell binding role, is often known as a metastasis suppressor protein [10,11]. N-cadherin, also belonging to the group of classical cadherins, is less commonly termed as CDH2 or non-epithelial cadherin, and does not exist in normal epidermis. Because of its abundant prevalence in muscle cells and fibroblasts, it is also known as neural cadherin. [12]. An altered expression of N-cadherin on cell surfaces or its de novo appearance reflecting mesenchymal phenotypes was documented in various human malignancies, such as prostate, breast, lung, liver and urothelial cancer, as well as in oral SCC [13,14,15]. So far, there have been no reports in the literature on the expression of N-cadherin in skin cancers. The aim of our study was to investigate the E- and N- cadherin expression, with a parallel assessment of Ki-67 expression in BCC and SCC, as it has not been investigated yet.

## 2. Materials and Methods

### 2.1. Tissue Samples

Paraffin samples of NMSC of patients from the University Centre of General Dermatology and Oncodermatology, Wroclaw Medical University were included in the present retrospective analysis. The study was approved by the Ethics Committee of Wroclaw Medical University (KB-250/2023). All hematoxylin–eosin-stained samples were independently assessed to confirm the diagnosis by two experienced histopathologists. Following this procedure, all samples were subjected to immunohistochemical staining. For the routine histological examination to establish the diagnosis, specimens were fixed in 10% neutral buffered formalin, processed by paraffin embedding technique and stained with hematoxylin–eosin (HE).

### 2.2. Immunohistochemical Staining

Immunohistochemistry was performed on 4-µm thick paraffin sections using Autostainer Link48 (Agilent, Santa Clara, CA, USA). Slides were deparaffined and epitope retrieval was carried out by treating the slides with EnVision FLEX Target Retrieval Solution (Agilent) (97 °C, 20 min; pH 9 for E-Cadherin and N-Cadherin and pH 6 for Ki-67) using PTLink Platform (Agilent). Then, slides were cooled in EnVision FLEX Wash Buffer (Tris-buffered saline solution containing Tween 20) (Agilent). Endogenous peroxidase was blocked using EnVision FLEX Peroxidase-Blocking Reagent (5 min; RT, Agilent) followed by a washing step with EnVision FLEX Wash Buffer. Afterward, primary antibodies against E-Cadherin (RTU, IR059, Agilent) and N-Cadherin (1:50, M3613, Agilent) against Ki-67 clone MIB-1 (RTU, IR626, Agilent) were applied for 20 min at RT. After washing in EnVision FLEX Wash Buffer, the sections were incubated with EnVision FLEX/HRP (horseradish peroxidase) secondary antibodies (20 min, RT) (Agilent). Subsequently, sections were washed in EnVision FLEX Wash Buffer and the substrate for peroxidase, diaminobenzidine (DAB), was applied for 10 min at RT. Finally, all the sections were counterstained with FLEX Hematoxylin (Agilent) for 5 min at RT, and dehydrated in ethanol alcohol (70%, 96%, 99,8%) and xylene. Subsequently, the slides were mounted in a Mounting Medium (Agilent). Primary antibodies (N-cadherin) were diluted in FLEX Antibody Diluent (Agilent).

### 2.3. Evaluation of Reactivity

Two experienced histopathologists to eliminate interobserver bias evaluated the immunohistochemical staining results with an optical microscope (Olympus BX 41 light microscope, Tokyo, Japan) under 400× magnification. Any disagreements were resolved by a constructive discussion. In all specimens of BCC and SCC, the expression of E-Cadherin and N-Cadherin manifested by the percentage of stained cells was assessed on a four-grade scale, with grades of 0%, 1–25%, 26–50%, and >50% stained cells, respectively, according to Remmele [16]. With regard to the evaluation of the antigen Ki-67, the percentage of positively stained cell nuclei was counted on a five-grade scale. The five grades were 0%, 1–10%, 11–25%, 26–50%, and >50% cells, respectively, demonstrating expression of a Ki-67antigen. The immunoreactivity evaluation of E and N-cadherin was assessed on a 4-point scale, as this is a scale dedicated to membrane reactions, whereas the 5-point scale (Ki-67) is used for nuclear reactions [16]. For both E-cadherin and N-cadherin, the whole specimen was evaluated, as is routinely practiced with regard to the membrane proteins. As far as the Ki67 antigen is concerned, at least three representative sites, the so-called “hot spots”, interpreted as sites with the highest number of positive cells were examined. The mean of observed fields was estimated and evaluated in all groups.

### 2.4. Statistical Analysis

The categorical variables were presented as absolute numbers and percentages. After assessment for normality using the Shapiro–Wilk test, numerical variables were presented as the median, with quartiles 1 and 3 as all variables demonstrating non-normal distribution. Direct comparisons were made between BCC and SCC. The Pearson’s chi-squared or Fisher’s exact tests were used to make the between-group comparisons for the categorical variables depending on the number of observed or expected values and, due to the non-normal distribution of all numerical variables, the Mann–Whitney U test was used for comparisons between numerical variables. The two-tailed *p*-value < 0.05 was considered statistically significant. STATISTICA 10 software (StatSoft Inc., Tulsa, OK, USA) was used for all calculations.

## 3. Results

The study population included 123 NMSCs, 73 BCCs and 50 SCCs. As seen in Table 1, the most common locations of BCC were eyelid (31.5%), ear (24.7%) and cheek (12.3%).

**Figure 1:**
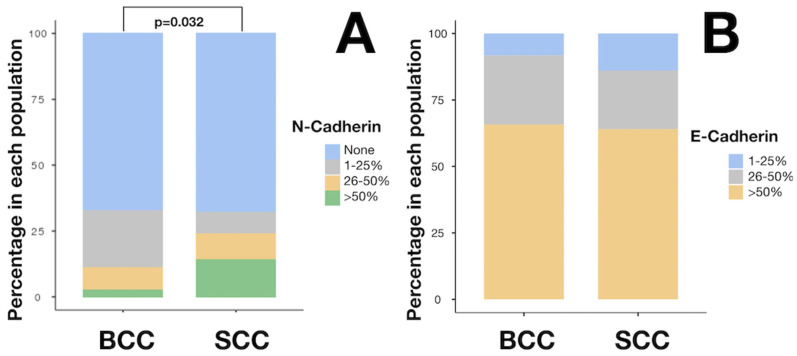
Levels of expression of N-cadherin (**A**) and E-cadherin (**B**) in BCC and SCC. Abbreviations: BCC—Basal cell carcinoma; SCC—squamous cell carcinoma.

The most common histological subtypes of BCC were nodular (39.7%) followed by infiltrating (9.6%) and superficial (6.9%). The most common anatomical locations of SCC were the ear followed by the cheek and scalp regions with the same 14.0% incidence in the last two locations, as seen in Table 1. The detailed expression of analyzed molecules is presented in Table 2 and Figure 1.

**Figure 2:**
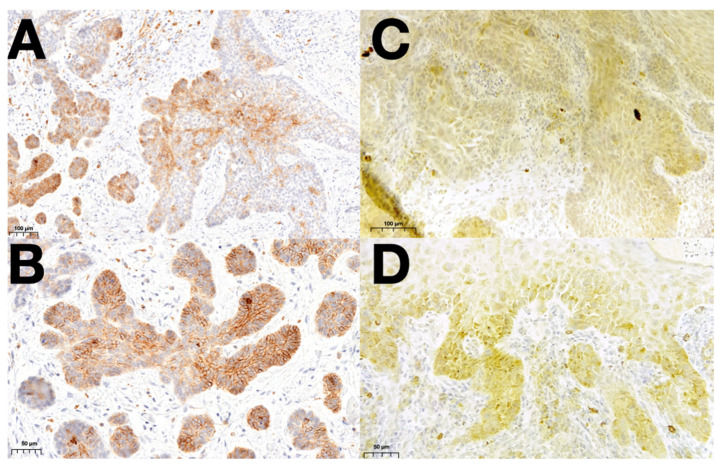
Representative patterns of N-cadherin staining of BCC in 200× (**A**) and 400× (**B**) magnification and of SCC in 200× (**C**) and 400× (**D**) magnification. Abbreviations: BCC—Basal cell carcinoma; SCC—squamous cell carcinoma.

A significantly higher N-cadherin expression was demonstrated in SCC compared to BCC (*p* = 0.032), as there were 14% of SCC cases with more than 50% cells expressing N-cadherin, 10% with 26–50% and 8% with 1–25% expression. The respective values for BCC cases were significantly lower, being 2.7% cases with expression above 50%, 8.2% with 26–50% and 21.9% with less than 25% expression of N-cadherin. The representative images of N-cadherin staining in the analyzed BCC and SCC specimens are presented in Figure 2.

**Figure 3:**
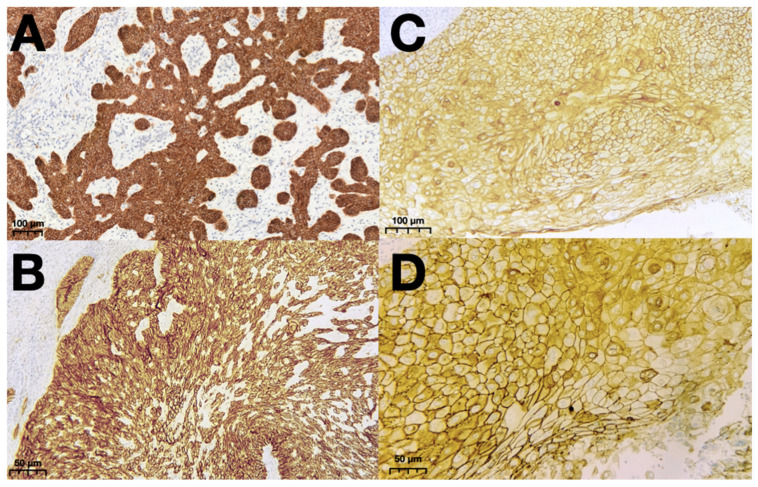
Representative patterns of E-cadherin staining of BCC in 200× (**A**) and 400× (**B**) magnification and of SCC in 200× (**C**) and 400× (**D**) magnification. Abbreviations: BCC—Basal cell carcinoma; SCC—squamous cell carcinoma.

The expression of E-cadherin was strong and preserved in both SCC and BCC. There were 22.0% SCCs with expression exceeding 26% and 64% cases with more than 50% expressing E-cadherin, whereas for BCC, these values were similar, namely 26.0% and 65.8%. Figure 3 demonstrates the exemplary E-cadherin staining of BCC and SCC.

**Figure 4:**
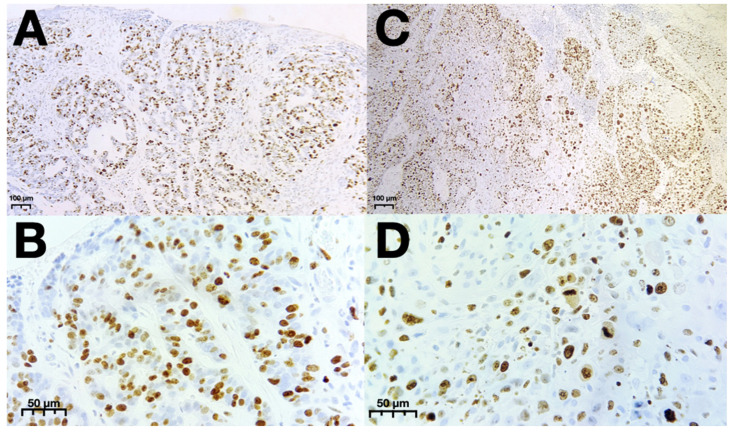
Representative patterns of Ki-67 staining of BCC in 200× (**A**) and 400× (**B**) magnification and of SCC in 200× (**C**) and 400× (**D**) magnification. Abbreviations: BCC—Basal cell carcinoma; SCC—squamous cell carcinoma.

No significant difference was observed between the two cancers with regard to E-cadherin. The Ki-67 expression in both skin cancers was very high. Regarding SCC, there were 28% cases with Ki-67 expression > 50% cells. Moreover, almost half of the SCC cases showed expression 26–50%, whereas in the case of the BCC, there were 30.1% samples with Ki-67 expression > 50% cells and 45.2% with expression 26–50%. In Figure 4, the example of Ki-67 staining in both BCC and SCC is presented.

**Figure 5:**
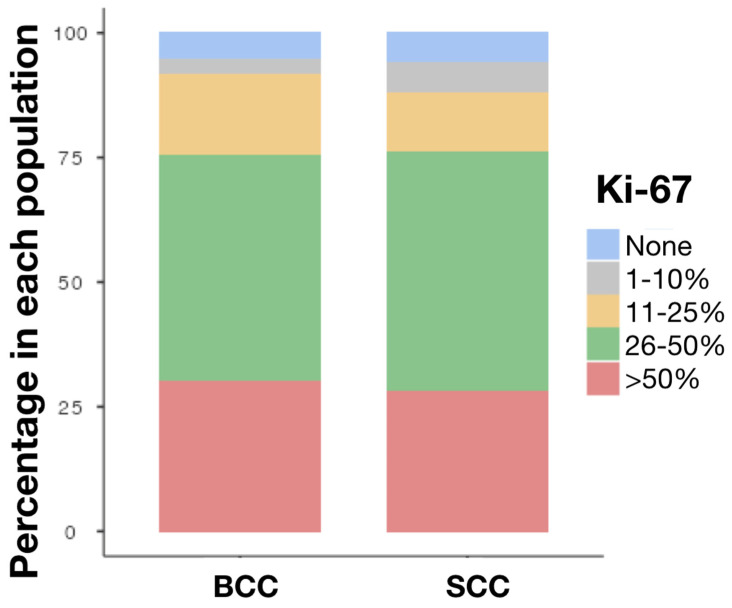
Levels of expression of Ki-67 in BCC and SCC. Abbreviations: BCC—Basal cell carcinoma; SCC—squamous cell carcinoma.

Since the Ki-67 expression results for both SCC and BCC were comparably high, as for E-cadherin, there was no statistically significant difference between the two cancers, as demonstrated in Figure 5.

## 4. Discussion

The results of our study indicate a higher expression of N-cadherin in SCC, when compared with BCC cases, without significant between-tumor difference in the expression of either E-cadherin or Ki-67. Both E- and N-cadherin are transmembrane glycoproteins belonging to classical cadherins, which are responsible for calcium-dependent cell–cell adhesions among keratinocytes, and also among keratinocytes and melanocytes [8,17]. On the membranes of the healthy epithelial cells, there is a high expression of E-cadherin, which is responsible for the maintenance of tight intercellular connections; therefore, this cadherin is often referred to as a metastasis suppressor protein [7,10]. In our study, E-cadherin was preserved and comparably highly expressed in both BCC and SCC. As in the process of distant metastasis formation, the breaking of intercellular junctions should occur at an early stage; it is not surprising that in both BCC and SCC, there was a high expression of E-cadherin with a relatively low metastatic potential. Our results are consistent with some of the literature reports that confirmed a preserved expression of E-cadherin in both BCC and SCC [18,19,20]. It should be noted that there are reports in the literature of reduced E-cadherin expression in both SCC and BCC, with a remark that such a reduction was more pronounced in tumor variants regarded as more malignant [21,22]. In another study, the authors proved a decrease in E-cadherin in both BCC and SCC with preservation of its high expression in SCC in situ [21]. Thus, despite the partially divergent results, it generally appears that the decrease in E-cadherin, if it occurs, tends to relate to more malignant histopathological subtypes of NMSC. Analyzing our result in the context of the above-mentioned articles, it appears that high E-cadherin expression may be a reasonable explanation for the generally low metastatic potential of both BCC and SCC. N-cadherin is widely expressed during embryonic life and is involved in the development and regulation of nervous tissue and the brain, heart and other organs. After the development period, only neurons, mesothelial cells, muscle cells, lens epithelial cells and fibroblasts express N-cadherin [12]. It is assumed that the N-cadherin expression on cells may indicate a phenotypic change from epithelial to mesenchymal and its presence may enhance migratory and invasive capabilities of tumor cells [12,23]. This process has been documented in many tumors, such as breast, lung, prostate and pancreatic cancer, as well as in melanoma and oral squamous cell carcinoma (OSCC) [13,14,15,24,25]. Studies on breast cancer cell lines have demonstrated that N-cadherin is upregulated in more invasive cancer cells. Moreover, N-cadherin may also facilitate a critical step in the passing of the vascular barrier by tumor cells (the so-called “extravasation”) and thus may directly contribute to tumor invasion [26]. Interestingly, it was proved that even when E-cadherin is preserved, N-cadherin can increase tumor cell motility [27].

So far, the only studies to evaluate the N-cadherin expression in any SCC have been performed in SCC located in the oral cavity. OSCC, as one of the most prevalent tumors of the head and neck region, also develops in the squamous epithelium, but has a much more aggressive course, a worse prognosis and more significantly frequent metastases than cutaneous SCC [28]. The higher malignancy of OSCC is one of the reasons why attempts are still being made to find both the explanation for the generally worse clinical course in this location and a factor that would make it possible to predict the degree of invasion. In one of the studies, an increase in the expression of N-cadherin was seen in 80% of OSCC cases after an immunohistochemical analysis. Moreover, a simultaneous reduction of E-cadherin expression was noted in 40% of the OSCC. The authors hypothesized that this might explain the relatively high aggressiveness of this cancer [29]. The authors of another study conducted on different histopathological grades of OSCC concluded that N-cadherin expression can be useful in predicting the state of tumor progression and for histopathological grading of the tumor because of evident expression of N-cadherin in OSCC with poor histological differentiation [30,31,32]. However, it must be noted that another study revealed that although a switch from E-cadherin to N-cadherin may play a critical role in cancer development and metastasis, the expression of cadherins did not appear to differ significantly considering the histological grade and invasion [33]. Despite slight variances in results, so far, all studies on OSCC have demonstrated the appearance of N-cadherin in OSCC cells. Thus, an acquisition of a mesenchymal phenotype manifested by N-cadherin expression might be an explanation for its unfavorable clinical course. To our knowledge, our study is the first one to analyze the expression of N-cadherin in cutaneous SCC. We have observed a very high expression of this adhesion molecule and noticed 14% of SCC cases with more than 50% cells expressing N-cadherin, 10% with 26–50% and 8% cases with less than 25% expression. When discussing the results in BCC, it should be emphasized that our study is also the first to investigate N-cadherin in this skin cancer. We documented the presence of N-cadherin, albeit the expression was not as high as in the SCC. An expression in more than 50% of cells was present only in 2.7% of BCCs, expression in 26–50% was demonstrated in 8.2%, and less than 25% in 21.9% of BCC cases, reaching a statistical significance when compared with SCC. However, in 68% of SCCs and 67.1% of BCC, there was no expression of N-cadherin. In our opinion, this result may reflect the relatively low metastatic potential of NMSC in comparison with other cancers. Moreover, it can be speculated that SCC is known as more aggressive with slightly higher rates of metastases compared to BCC, and may have a higher potential to acquire mesenchymal characteristics reflected in the acquisition of N-cadherin on its cells. Beyond adhesion molecules, there are many factors, the expression of which may be important in the assessment of the progression of cancer tissue. Among them, one of the most important is the Ki67 antigen. This high molecular weight protein present during all active phases of the cell cycle is considered a marker of cellular proliferative activity; therefore, it may reflect the biological behavior of tumors [28]. High expression of this antigen has been shown to correlate with metastatic potential and recurrence of many cancers [34,35]. In our studied cases of NMSC, there was a high expression of Ki67. This confirms that the analyzed cancers proliferate as rapidly as many other solid tumors. Moreover, we observed no differences between SCC and BCC when comparing Ki67 expression. When analyzing the available literature, it should be emphasized that although there are only a few articles comparing SCC and BCC in terms of Ki67 expression, the results are divergent. Some of them, as in our study, describe no difference in Ki67 expression in the two cancers [36,37,38,39], and some confirmed higher Ki-67 expression in SCC [40,41,42]. We are aware of the limitations of our study, which are primarily lacking a simultaneous examination of the expression of “cadherin switch” regulating factors; for example, ZEB1/2, Twist and Snail, which can affect the expression of cadherins [43]. Additionally, while our study focuses on the expression of adhesion molecules in NSMC, the lack of data on the possible association with genetic findings, including the mutational spectrum of tumors, might be considered another limitation of our study. In addition, long storage time for tissue sections before staining for some antibodies may have led to a loss in antigen reactivity [44]. Another limitation of our manuscript is the assumption of the grading system for immunohistochemical expression, which precludes the retrospective calculation of the exact percentage of positive cells. Moreover, the number of studied cases might not allow for generalization to a broader spectrum of tumors, including less represented subtypes of both SCC and BCC. Finally, the aim of the present study was to focus on the levels of adhesion molecules, without correlation to clinical data, including the long-term prognosis of these tumors.

## 5. Conclusions

In our study, no significant differences in the expression of E-cadherin and Ki-67 between BCC and SCC were identified. However, a higher expression of N-cadherin SCC might highlight the more pronounced mesenchymal transformation of SCC cells, potentially contributing to a worse prognosis and higher rate of metastases compared with BCC. Further studies are needed in this area to confirm our findings.

## Figures and Tables

**Table 1:** The anatomical locations of BCC and SCC cases, and the histological subtypes of BCC.

Anatomical Locations of BCC Cases	Total Number, n = 73	Histological Subtypes of BCC Cases	Total Number with Histological Analysis, n = 67	Anatomical Locations of SCC Cases	Total Number, n = 50
Eye/Eyelid, n (%)	23/73 (31.5%)	Nodular, n (%)	29/67 (43.3%)	Lip, n (%)	4/50 (8.0%)
Ear, n (%)	18/73 (24.7%)	Basosquamous, n (%)	4/67 (5.9%)	Ear, n (%)	7/50 (14.0%)
Nose, n (%)	7/73 (9.6%)	Morpheaform, n (%)	1/67 (1.5%)	Nose, n (%)	5/50 (10.0%)
Temple, n (%)	2/73 (2.7%)	Infiltrating, n (%)	7/67 (10.4%)	Temple, n (%)	6/50 (12.0%)
Forehead, n (%)	3/73 (4.1%)	Superficial, n (%)	5/67 (7.5%)	Forehead, n (%)	5/50 (10.0%)
Cheek, n (%)	9/73 (12.3%)	Micronodular, n (%)	2/67 (3.0%)	Cheek, n (%)	7/50 (14.0%)
Other locations, n (%)	11/73 (15.1%)	Others, not specified, n (%)	19/67 (28.4%)	Scalp, n (%)	7/50 (14.0%)
				Other locations, n (%)	8/50 (16.0%)

**Table 2:** Percentage of N-cadherin, E-cadherin and Ki-67 in the assessed specimens of BCC and SCC.

	BCC, n = 73	SCC, n = 50	*p*
N-cadherin, no expression, n (%)	49 (67.1%)	34 (68.0%)	0.032
N-cadherin, 1–25%, n (%)	16 (21.9%)	4 (8.0%)	
N-cadherin, 26–50%, n (%)	6 (8.2%)	5 (10.0%)	
N-cadherin, >50%, n (%)	2 (2.7%)	7 (14.0%)	
E-cadherin, 1–25%, n (%)	6 (8.2%)	7 (14.0%)	NS
E-cadherin, 26–50%, n (%)	19 (26.0%)	11 (22.0%)	
E-cadherin, >50%, n (%)	48 (65.8%)	32 (64.0%)	
Ki-67, none, n (%)	4 (5.5%)	3 (6.0%)	NS
Ki-67, 1–10%, n (%)	2 (2.7%)	3 (6.0%)	
Ki-67, 11–25%, n (%)	12 (16.4%)	6 (12.0%)	
Ki-67, 26–50%, n (%)	33 (45.2%)	24 (48.0%)	
Ki-67, >50%, n (%)	22 (30.1%)	14 (28.0%)	

Abbreviations: BCC—Basal cell carcinoma, SCC—Squamous cell carcinoma, NS—Non significant.

## Data Availability

All data may be available on request from the authors.
